# Social Security Measures of Reproductive Health Among Adolescents in India: A Narrative Review

**DOI:** 10.7759/cureus.28546

**Published:** 2022-08-29

**Authors:** Alka Mahobia, Sonali G Choudhari

**Affiliations:** 1 Epidemiology and Public Health, Jawaharlal Nehru Medical College, Datta Meghe Institute of Medical Sciences, Wardha, IND; 2 Epidemiology and Public Health/Community Medicine, Jawaharlal Nehru Medical College, Datta Meghe Institute of Medical Sciences, Wardha, IND

**Keywords:** social security, social security measures, adolescence, adolescents, rmnch+a, india, sexual health, reproductive health

## Abstract

Adolescence is one of life's most complex stages, associated with unique reproductive health requirements. Adolescents have individual sexual and reproductive health requirements (whether or not they are sexually active or married). Young and developing children have limited knowledge and awareness of the biological changes that occur through adolescence and the illnesses that affect them. We aim to discuss adolescent problems related to reproductive health, estimate the social security measures and associated factors among adolescents, and find why healthcare services are gravely limited in terms of accessibility and availability. This paper was based on previously available data through multiple sources and literature searches on PubMed, Research Gate, Sci-Hub, and Google Scholar. Adolescent health programs are currently fragmented, with no comprehensive program addressing all of the needs of adolescents. The major barriers are a lack of reliable information, a lack of proper guidance, parental ignorance, a lack of skills, and inadequate services from the healthcare system. We need a multidimensional approach that addresses all young adults' health issues, focusing on reproductive health, social security, mental well-being, and interventions involving communication toward a healthy lifestyle.

## Introduction and background

India is home to more than 243 million adolescents, who account for almost 20% of the country’s population [1]. Reproductive health is vital to the physical, mental, and social well-being in all things relevant to the reproductive system, its functions, or processes, not simply the absence of illness or infirmity [2]. The new entry phase from childhood to adulthood is marked by significant physical, psychological, and social behavioral changes that may ruin their lives [3]. Adolescents are susceptible to a wide range of severe health conditions like violence, mental health, alcohol and drug abuse, unwanted pregnancies, unsafe abortions, early pregnancy and childbirth, injuries, sexually transmitted infections (STIs) including HIV/AIDS, and all forms of sexual violence. In some member states, early marriage and childbirth are linked to higher maternal morbidity and mortality and perinatal and neonatal mortality in adolescents [4]. Social security is a safety net, a warning sign, and a transcendent force. Its analysis is to determine what long-term change is to be achieved. To effectively respond to adolescent health and financial requirements, it is crucial to situate teenage development and well-being needs within vibrant sociological, cultural, and economic data. To achieve this, the Indian government established national programs such as Adolescent Reproductive and Sexual Health (ARSH) in 2005 and Rashtriya Kishor Swasthya Karyakram (RKSK) in 2014 [4]. In 2014, in line with the new National Reproductive, Maternal, Newborn, Child and Adolescent Health (RMNCH+A) Strategy's commitment to a continuum of care approach, the Ministry of Health and Family Welfare (MoHFW) replaced the ARSH strategy with RKSK [5].

Adolescence is a period of biological growth and development when the transition from childhood to adulthood occurs [6]. Both adolescent girls and boys have limited links to knowledge about issues that affect them, as well as fewer options to develop the skills needed for active participation. Adolescent girls, specifically, are susceptible on multiple levels, which can result in virulent social norms that diminish the value of girls, limiting their right to free movement and making decisions that impact their work, education, wedding, and social relationships [7]. Previous research indicates that adolescents' lack of adequate knowledge about STIs and individuals' knowledge levels improve significantly due to the instructional model. At this age, they become more interested in sexuality and start to show interest in sex in the opposite gender. The mainstream press also plays a crucial role in revealing people to internet pornography, leading to them being sexual assault perpetrators [1]. Positive social health and physical health add to adolescents' general health. Adolescents are becoming more susceptible to sexual, physical, and abusive language. For an extended period, no proper system has been organized as a framework for controlling the social needs of young people. Poor reproductive, maternal, newborn, and child health and nutrition (RMNCH&N) outcomes remain a major public health concern in the country. For instance, nearly half of the pregnant women are anemic as per the National Family Health Survey (NFHS) 2015-2016, and it has only marginally declined from NFHS 2005-2006 (58%) [8].

Today, adolescents account for 16% of the world's population, with India having the nation's most prominent adolescent citizens [9,10]. In India, at least 42% of girls prefer cloth sanitary pads to disposable sanitary napkins [7]. It is not shocking that only 26% of adolescent girls with heavy menstrual bleeding sought medical attention [9]. In total, 64% of girls have at least one menstrual-related issue [11]. Approximately 9% of rural adolescent girls and 5% of urban adolescent girls aged 15-19 years had given childbirth. In total, 3% have gotten their first kid, and despite widespread understanding of modern contraception (>90%), one-fifth of adolescent girls have used a contemporary method of contraception [12]. Adolescents lack adequate sexual reproductive health awareness and knowledge, which would be a source of worry [13]. The majority of sexual activities begin throughout adolescence; 3% of adolescent males and 8% of adolescent females had intimate relations well before the maturity level of 15, 1% of females and 63% of males aged 15-19 years had elevated sex with a non-marital, non-cohabiting companion, and 31% of adolescent males and 20% of adolescent females were using a contraceptive [14]. Adolescents received less skilled birth treatment than all women and girls combined (75-81%). At the same time, the proportion of adolescent girls receiving postpartum care was similar to all women and girls, i.e., around 65% [9]. Adolescent girls did receive less maternal and neonatal treatment globally than all women and girls merged (75-81%). At the same time, the percentage of teenage girls receiving postnatal care has been similar to those of women and girls, i.e., about 65% [15]. The prevalence of self-reported reproductive tract infection (RTI) symptoms among Indian women is 11-18% in nationally representative studies [16,12]. According to the World Health Organization (WHO), more than one million people worldwide acquire STIs. The WHO estimates that 500 million new cases of one of four curable STIs (chlamydia, gonorrhea, syphilis, and trichomoniasis) occur worldwide [17]. Anemia is a major public health problem globally, affecting over 1.9 billion people [18].

Reproductive and sexual health

Adolescents face several sexual and reproductive health issues. According to the NFHS-3 data, 2.7% of boys and 8% of girls had their first sex act well before the age of 15, and the number of sexual activity takes place within the context of marital relationships, which results in rapid childbirth associated with social pressure [7,19]. The percentage of adolescents is shown in Table 1.

**Table 1:** Percentage of adolescents in the top and bottom five states Adapted from [7].

Top five states	Adolescents (%)	Bottom five states	Adolescents (%)
Uttar Pradesh	24.5	Kerala	16.3
Rajasthan	22.9	Tamil Nadu	17.2
Uttarakhand	22.5	Karnataka	18.9
Bihar	22.5	Maharashtra	19.0
Jharkhand	22.2	Andhra Pradesh	19.3

Even though 94% of girls aged 15-19 years seem to be conscious of contraceptive methods, only 23% of married girls and 18% of sexually active unmarried females in this age category used a form of contraception at least once. All three NFHS have nearly equal prevalence in pregnant and parented adolescents (59.1%, 59.8%, and 58.2%, respectively), and the percentage of the age of the first pregnancy among many adolescents is increasing steadily (11.7%, 12.4%, and 14.4%, respectively). Sexual relations and low condom use are the causes of this trend [7]. The status of reproductive and sexual health is shown in Table 2.

**Table 2:** Status of reproductive and sexual health of Indian adolescents (UNICEF and NFHS3) Adapted from the United Nations International Children's Emergency Fund (UNICEF) [9] and the National Family Health Survey 3 (NFHS3) [20]. STI: sexually transmitted infection.

Factors	Boys (%)	Girls (%)
Before the age of 15, the sexual debut	2.7	8.0
Awareness of contraceptive methods (15-19)+	96.0	94.0
Control method ever used+	29.4	40.4
Birth by age 18 (2008-2012)+	-	21.7
Condom use during the first time	19.0	3.0
Comprehensive understanding of HIV in adolescents	34.5	18.6
STI symptoms in youngsters	10.8	10.5
HIV prevalence among adolescents	0.01	0.07

## Review

This paper discusses the adolescent reproductive health problem and estimates the social security measures and their associated factors among adolescents in India. Our report was based on previously available domestic and worldwide literature discovered through a thorough search of multiple sources. We search various government portals and websites for current related data and literature in PubMed, ResearchGate, Sci-Hub, and Google Scholar. The goal was to create a policy change, raise knowledge and understanding among the public, improve adolescent healthcare services and social security, and meet their needs and expectations. In India, reproductive health and associated sexual illnesses are significant but understudied health and safety issues. However, as a country, India does not currently have an adolescent health policy, requiring the creation of a national adolescent healthcare policy.

Our review suggests that sexual and reproductive health (SRH) education, counseling, and contraceptive availability effectively increase adolescent knowledge related to sexual health, menstruation, contraceptive use, and decreasing adolescent pregnancy.

Significant problems associated with reproductive health are as follows: (i) overpopulation: explosion in population causes a scarcity of every basic need and hence affects the well-being of reproductive health. (ii) Sex education: due to a lack of sex education, people are not concerned about safe and hygienic sexual practices. (iii) Adolescence-related changes: the changes in adolescents can lead to sex abuse and affect reproductive health. (iv) Sexually transmitted diseases: sexually transmitted infections can affect reproductive health. (v) Sex abuse and sex-related crimes: sex abuse can cause physical injury, unwanted pregnancy, vaginal discharge, pelvic pain, etc.

Discussion

Government Programs

For social security and betterment of adolescent health, the government took the next step, and the ARSH strategy was developed in 2005 by the Indian government's MoHFW as part of the National Rural Health Goal and Human Development and Children's Health Program to "broaden the definition and media attention of family assistance programs." ARSH initiatives were included in the Reproductive Child Health-II (RCH-II) and performance improvement plans (PIPs) of several states and union territories to aid in accurately and effectively implementing program activities. Through a fundamental bundle of preventive, promotional, and curative services, the ARSH is also a specific health feature for all adolescents [21]. The ARSH strategy primarily employed a treatment center strategy that concentrated on building the utilization of redirecting resources of healthcare personnel and also realigning brand recognition and establishing health services as adolescent-friendly health centers. The method likewise formed fair standards for improving the quality of services and guidance for implementing them. While generating demand was less concentrated, a few nations supplemented the strategic plan with initiatives to boost provider knowledge and sensitize societies.

In 2014, the ARSH strategy was replaced by RKSK because of the new RMNCH+A strategy's contribution to an approach to the delivery of healthcare. SRH has been pushed aside in favor of noncommunicable diseases, nourishment, psychological health, injuries, substance misuse, and abuse. It uses hospital and social service delivery models and generates demand initiatives. RKSK's execution is currently in progress in the country with a particular emphasis on 213 districts [22].

Menstruation

The onset of menstruation is one of the most significant transformations that girls go through during their adolescent years. Menstrual hygiene management (MHM) and practices by adolescent females of low and middle-income countries (LMICs) are a severe concern [23]. Hygiene-related practices during menstruation can increase the risk of developing reproductive tract infections [24]. Although menstruation is a normal part of life and is associated with several myths and misunderstandings that might negatively affect health, menstruation is still seen as repulsive or dirty in Indian society [25]. MHM is a severe problem in India for school-aged teenagers due to a lack of safe, sanitary facilities and limited or no pure hygiene products; many girls drop out of school due to a shortage of menstrual hygiene products and services [24]. Menstrual abnormalities and disorders are frequently linked to physical, mental, social, psychological, and reproductive issues, affecting adolescents' daily lives and their families live through various psychosocial problems such as anxiety [26]. Maintaining good hygiene for women during menstruation is of considerable importance, especially regarding increased vulnerability to reproductive tract infections [27].

Contraceptives

The primary reason for low contraceptive use may be inadequate access to contraceptive knowledge and services; other reasons could be early marriage and childbearing, which were associated with everyday contraceptive use and lower education [28]. The high prevalence of reproductive tract infections is due to fewer contraceptives in India. The current use of contraceptives was reported by 26.4% of indigenous women in the reproductive age group [29]. Knowledge of contraceptives underpins their use. The above-average knowledge scores were higher among the educated and aligned with the above-average knowledge of emergency contraceptives [29]. Contraceptive-induced menstrual changes (CIMCs) affect contraceptive users' lives positively and negatively [30].

Adolescent-Friendly Health Clinics

As part of its facility-based approach, RKSK highlights the importance of reinforcing adolescent-friendly health clinics (AFHCs) [22]. Adolescents face health issues that adult and pediatric doctors are often unready to address. Communities must address their specific needs to shield adolescents from threats such as disease, sexually transmitted diseases, unintended pregnancy, HIV transmission, and alcohol and drug abuse, and governments should invest in the institution of adolescent-friendly healthcare services in health facilities, clinics, and youth centers. It is critical to create a welcoming, private space where adolescents can feel at ease and obtain prescriptions and counseling to realize the right to receive healthcare services [31].

Sexual and Reproductive Health

SRH is the most vital component in the adolescent phase of life. The behavioral pattern established during this phase determines the current health status and the risk of developing chronic ailments later in life [12]. The state should stand committed to ensuring good, affordable, and accessible adolescent health services [32]. High-risk sexual behavior is a broad term covering early sexual activity, especially before 18 years of age, and includes unprotected intercourse without male or female condom use except in a long-term, single-partner (monogamous) relationship, unprotected mouth-to-genital contact except in a long-term monogamous relationship, having multiple sex partners, and having a high-risk partner [33]. Women of reproductive age are at high risk for anemia and iron deficiency due in part to blood losses during menstruation and increased iron requirements during pregnancy [34].

Pubertal Behavior

Understanding these puberty-specific behavioral changes represents an essential dimension of normal development in adolescence; it also has broad clinical and social policy relevance. Interactions between these motivational tendencies and the social contexts that amplify these tendencies are relevant to understanding the health paradox of adolescence [35]. Reproductive types of adolescent health problems appear heterogeneous; on the other hand, most of these health consequences reflect difficulties with controlling emotion and behavior. Interpersonal violence among youth, ranging from minor acts of bullying to severe forms of homicide, contribute significantly to the burden of premature death, injury, and disability; it has not just affected adolescents but also their families, friends, and communities [36].

Recommendations

Identifying the adolescents' perceived needs during planning is critical, and services must be of good quality. It is also crucial to raise adolescents' understanding of the importance and need for the services and encourage them to use them. Steps should be taken to postpone the marriage age through advocacy, advice, and stringent measures adherence to the legislation. Adults should be informed to avoid young marriage and adolescent childbirth and the problems that come with it. All primary health centers (PHCs) must be capable of providing AFHS beyond the conventional times. A trendy wing can be established to classify adolescents at the tertiary and secondary care levels.

Community involvement should be encouraged in mobilizing adolescents to develop life skills and they should participate actively in community programs. Government and non-government organizations have taken various steps to raise concerns about the consequences of unchecked population growth and social ills such as sexual misconduct and sex-related crimes. The introduction of sex education in schools and universities is another proceed providing the appropriate evidence to the youth. Amniocentesis is used to determine sex legally and should be prohibited because it increases female feticide. To achieve reproductive health, massive child immunization programs such as human papillomavirus (HPV), tetanus, diphtheria, and whooping cough (pertussis) (Tdap), meningococcal disease (MenACWY), hepatitis A (HepA), hepatitis B (HepB), polio (inactivated poliovirus vaccine), measles, mumps, and rubella (MMR), and chickenpox (Varicella) should be implemented.

## Conclusions

Adolescents were very limited and associated with their attitudes and knowledge of SRH issues. Our findings identify the need to improve SRH communication to adolescents and provide correct awareness in a positive way. To accomplish excellent and healthy reproductive health for every adolescent and provide social security in society, we need a multidimensional approach that addresses all young adults' health issues, with a focus on mental well-being, interventions involving communication toward a healthy lifestyle, and a positive social setting in which to learn life skills. Therefore, policymakers should involve multiple sectors like health, education, and youth services to provide many sources of information to change the negative attitudes toward SRH communication with adolescents. Adolescent-friendly clinics must be broadly accepted throughout India to achieve full coverage. Regular adolescent screening is an effective tool for controlling existing illnesses and tracking the occurrence of any sexual and reproductive diseases. Encourage and necessitate adolescents in the choice that impacts them, and use them as each change to grow into productive adults. Giving such a chance to adolescents to grow allows them to build a secure, joyful, wholesome, and constructive society in the future.
